# Effect of herbal tea on glycemic control in patients with type 2 diabetes: Protocol for a systematic review and meta-analysis

**DOI:** 10.1097/MD.0000000000018346

**Published:** 2019-12-16

**Authors:** Boxun Zhang, Rensong Yue, Xiaoying Huang, Ying Wang, Yayi Jiang, Jiawei Chin

**Affiliations:** a Hospital of Chengdu University of Traditional Chinese Medicine; b Chengdu Qingyang Hospital of Traditional Chinese Medicine, Chengdu, China.

**Keywords:** herbal tea, meta-analysis, protocol, systematic review, type 2 diabetes

## Abstract

**Background:**

Type 2 diabetes (T2D) is a significant health concern worldwide, and good glycemic control is the basis of avoiding disease progression. Herbal tea, as a convenient and effective medication method, has gained popularity among many diabetic patients. However, there are no systematic reviews or meta-analyses to evaluate the clinical efficacy of herbal tea on T2D.

**Methods:**

Four English electronic databases and 4 Chinese electronic databases were searched for randomized controlled trials (RCTs) meeting inclusion criteria; Clinical trials were searched to explore the relevant unpublished data. Fasting blood glucose and glycated hemoglobin will be measured as primary outcomes. Secondary outcomes include 2-hour postprandial blood glucose, fasting insulin, and homeostasis model assessment-insulin resistance. The heterogeneity of data will be investigated by Chi-square and *I*^2^ test; subgroup analysis and sensitivity analysis will be conducted to explore the sources of heterogeneity; funnel plot will be used to evaluate publication bias; finally, we will use grading of recommendations assessment, development, and evaluate system method to evaluate the quality of evidence. Merging analysis of data will be performed using Rev Man 5.3 software.

**Results:**

The results will be published in a peer-reviewed journal.

**Conclusions:**

The systematic review will confirm whether herbal tea consumption is benefit to the glycemic control in patients with T2D.

**PROSPERO registration number:**

CRD42019129863.

## 1 Introduction

Globally, diabetes has become a significant health concern in the past decades. Data from the International Diabetes Federation indicate that approximately 425 million adults (aged 20–79 years) have this disease, and US$673 billion was spent to improve this situation.^[1]^ Accounting for over 90% of diabetes cases, type 2 diabetes (T2D) is a metabolic disease characterized by insulin resistance and impaired insulin secretion.^[2]^ Although researchers have formulated treatment strategies for T2D from multi-dimensional perspectives, such as pharmacologic therapy, health education, exercise, and nutrition,^[3]^ the sharply increasing morbidity and socio-economic expenditure are still not effectively curbed.^[4]^ It is believed that good glycemic control is the basis to delay or even avoid diabetes-related complications, and there are countless researchers making great efforts to achieve this goal.^[5]^

Herbal medicine has been used to treat diabetes for thousands of years, and in recent decades, several studies have confirmed that herbal medicine has the characteristics of multi-ingredient, multi-target, and multi-pathway.^[6]^ On the one hand, it can exert direct therapeutic action by repairing damaged islet cells and improving insulin sensitivity; on the other hand, it can also potentially prevent diabetes-related complications through holistic and dynamic regulatory mechanisms.^[7,8]^

As a convenient and effective medication method, herbal tea has gained popularity among many diabetic patients and other health-conscious consumers.^[9]^ In general, herbal tea is prepared from the leaves, flowers, and fruits of herbal medicines.^[10]^ Similar to green tea, these raw materials are often packed in tea bags, and users only need to brew them in boiling water. As the traditional and fashionable beverage, many different herbal teas have been consumed worldwide. Bioactive compounds contained in herbal teas are variable according to different species and phenolic compounds, flavonoids, coumarins, alkaloids are common and significant.^[11]^ Animal experimental studies have confirmed that herbal tea can be used to treat T2D through anti-inflammatory and antioxidant effects, inhibiting α-glucosidase and other pathways.^[12]^

Herbal tea is the combination of herbal medicine and traditional tea. It is believed that, as the second most commonly consumed beverage, tea not only brings relaxation and enjoyment to people's lives but also treats and prevents some diseases.^[13]^ Evidence from some original studies and meta-analyses has indicated that habitual tea drinking can reduce the incidence of T2D and the serum glucose level of diabetic patients^[14,15]^; however, these conclusions are controversial.^[16,17]^ Over the past 2 decades, studies have found that drinking herbal tea with hypoglycemic activity may have a better therapeutic effect than common teas for T2D individuals, and with the deepening of the research, some clinical randomized controlled trials (RCTs) have been published. However, there are no systematic reviews and meta-analyses to evaluate their efficacy. Thus, the objectives of our study are:

(1) to collate the evidence for the clinical efficacy of herbal teas in the treatment of T2D; and
(2) to conduct a meta-analysis to determine whether herbal tea is more beneficial in the glycemic control of T2D than in the control groups.

## 2 Methods

### 2.1 Study registration

This systematic review and meta-analysis protocol have been registered on the international prospective register of systematic review (PROSPERO) and the registration number is CRD42019129863. This systematic review and meta-analysis protocol is reported according to the preferred reporting items for systematic reviews and meta-analysis protocols checklist.^[18]^

### 2.2 Inclusion and exclusion criteria

#### 2.2.1 Study design

Only RCTs will be eligible for our research, the studies of non-RCT clinical trials, animal experiments, comments, reviews, and real-world study will not be included.

#### 2.2.2 Participants

The research subjects were definitely diagnosed with T2D and did not have serious DM-related complications, and there will be no limitation about age, region, gender, and other factors.

#### 2.2.3 Interventions and comparators

The experimental group ingested herbal tea as the major adjunct therapy, and the intervention measures were tea bags containing 1 or more dry herbal powders or aqueous beverages mainly derived from herbs; there are no restrictions to the measures in control group.

#### 2.2.4 Outcomes

Primary outcomes are fasting blood glucose (FBG) and glycated hemoglobin (HbA1C). Secondary outcomes will include 2-hour postprandial blood glucose (2-h PBG), fasting insulin (FINS) and homeostasis model assessment-insulin resistance (HOMA-IR).

### 2.3 Study search

The following electronic resource databases will be searched from their inception: PubMed, the Cochrane Library, Embase, Web of Science, China National Knowledge Internet, Chinese Biomedical Literature Database, Wanfang, and Chinese Scientific and Technological Periodical Databases; Clinical Trials will be searched to explore the relevant unpublished data; the references of relevant systematic reviews that have been published previously will be manually searched to avoid omission. All the searches will use a combination of medical subject headings terms and free-text words; the final search strategy of each database was determined by multiple searches and modifications. There will be no restrictions on the publication year and language. The search strategy for Pubmed list in Table 1.

**Table 1:**
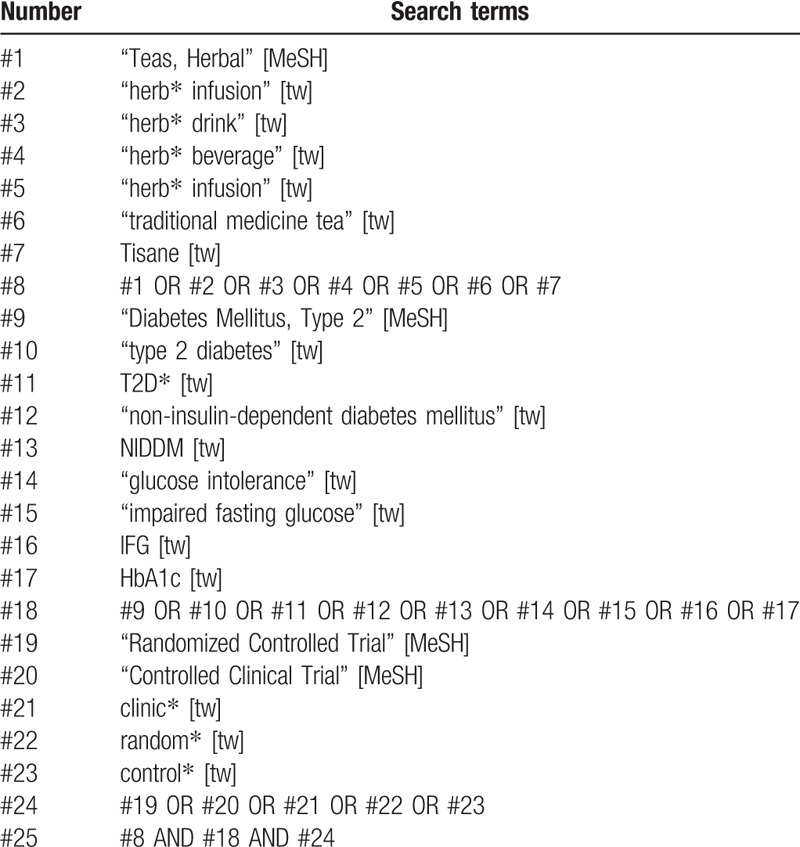
Search strategy for PubMed.

### 2.4 Study selection

**Figure 1:**
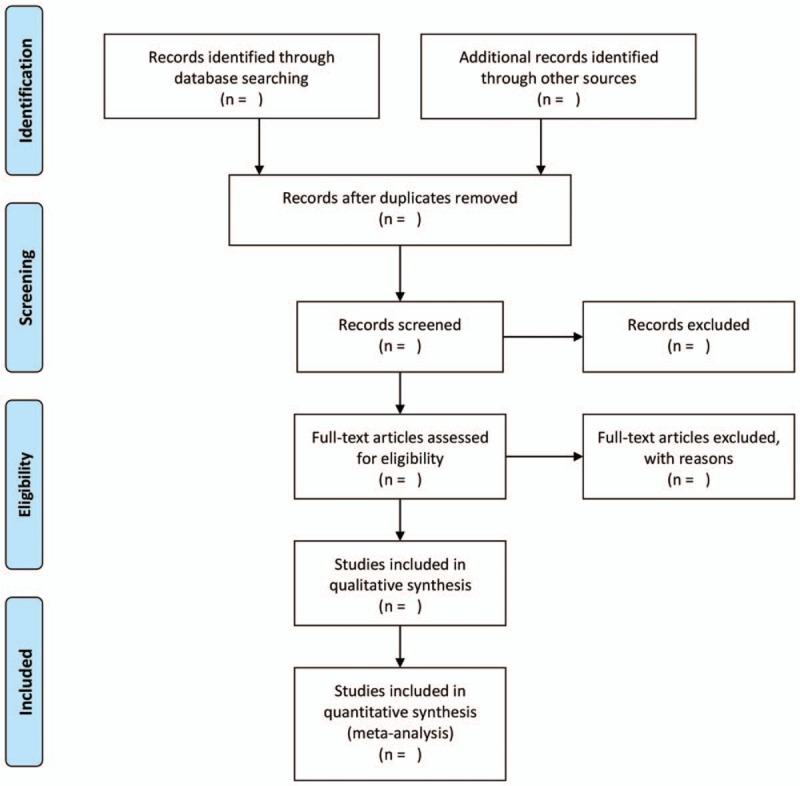
Flow chart of study selection.

EndNote X9 software will be used to manage the retrieved articles. After removing duplicate studies, 2 authors independently will screen the selected articles according to their titles and abstracts. After the preliminary assessment, we will continue to investigate the full text of the selected articles based on the inclusion and exclusion criteria. Regarding the differences arising in these processes, the third author will investigate independently and provide the final evaluation. We will use a flow chart to show the whole process of study selection (Fig. 1).

### 2.5 Data extraction

To summarize the data more clearly and comprehensively, we will transcribe the main information of the article into a concise form. The content included the title, author (year), country, sample size, basic information of participants, trial design, intervention, control measures, and outcomes. If the data or methodological details we need cannot available from the article, we will try to contact the corresponding author for more information.

### 2.6 Risk of bias assessment

Two authors will independently assess the methodological quality according to the guidance in the Cochrane Handbook for Systematic Reviews of Interventions (Version 5.2.0) based on 7 items: random sequence generation; allocation concealment; blinding of participants and personnel; blinding of outcome assessment; incomplete outcome data; selective reporting and other sources of bias. Each item was judged as being either “high risk” or “low risk” or “unclear risk” of bias. Discrepancies in the interpretation were resolved by an independent review by the third author.

### 2.7 Data analysis

RevMan 5.3 statistical software (Cochrane Collaboration, Oxford, UK) will be used for the meta-analysis. Because the data involved in this study were all continuous variables, the mean difference or standardized mean difference with 95% confidence interval was used. Chi-square distribution test and *I*^2^ statistic will be used to analyze the heterogeneity of included studies. *P* > .1 and *I*^*2*^ < 50% indicate that there is a acceptable heterogeneity, and a fixed-effects model will be applied; *P* ≤ .1 and/or *I*^2^ ≥50% suggest that there exist a significant heterogeneity and a random-effects model will be used. If quantitative synthesis is not appropriate due to substantial heterogeneity, only qualitative analysis will be performed.

### 2.8 Subgroup analysis

If there exists a significant heterogeneity, subgroup analysis will be performed to explore the sources. The following factors may be considered: types of herbal tea, regions of herbal tea, dosage, duration of treatment, measures in control groups, geographical area of patients, blood glucose value in baseline point.

### 2.9 Sensitivity analysis

To assess the stability of results and the sources of heterogeneity, we will perform the sensitivity analysis. Specifically, we will remove the data of a certain study 1 by 1, and then compare the recombined data with the original data. We will focus on the changes of merged size effects and heterogeneity.

### 2.10 Publication bias assessment

If there are enough original studies (≥10), publication bias will be evaluated by a funnel plot. Symmetrical funnel plot indicates low publication bias, otherwise, high publication bias.

### 2.11 Summary of finding tables

At last, 2 researchers will use the grades of recommendation, assessment, development, and evaluation (GRADE) method to evaluate the quality of evidences. After the assessment of certainty assessment, number of patients, effect, certainty and importance, GRADE system will define the level of evidence as “high,” “moderate,” “low” or “very low.”

### 2.12 Ethics and dissemination

Since meta-analysis is a secondary study of the published data, ethical approval is not needed. The results will be published in a peer-reviewed journal.

## 3 Discussion

Herbal tea is a common beverage widely consumed in many countries that are derived from years of experience in the struggle against disease by ancestral humans and is a valuable resource that needs in-depth study.^[9]^ Generally, the herbs used for herbal teas are safe, effective, and taste good. Compared with the common types of tea (black tea, oolong tea, green tea, and white tea), herbal tea often have more advantages in the prevention and treatment of chronic diseases such as T2D.^[10]^ In fact, whether herbal medicine and tea have adjuvant therapeutic effects on T2D has been a hot topic of widespread concern. Liu et al^[19]^ and Suksomboon et al^[20]^ have conducted a meta-analysis on the hypoglycemic effects of herbs in 2004 and 2011, respectively; the results showed that herbal medicine may improve glycemic control in T2D, while the conclusion deserves further examination in high-quality trials. Additionally, most studies are focused on a certain herb or herbal derivatives, such as ginseng,^[21]^ aloe vera,^[22]^ berberine,^[23]^ silymarin,^[24]^ and most have indicated that herbal medicine has a positive therapeutic effect on T2D. Tea, as the second most popular beverage worldwide, is also believed to regulate glycometabolism. A meta-analysis conducted by Li et al^[15]^ confirmed tea or tea extraction could maintain a stable fasting blood insulin level and reduce waist circumference, but there were no statistically significant differences in other indicators such as FBG; another study^[17]^ from Jinyue Yu and colleagues suggested no evidence to support that the consumption of green tea can effectively benefit glycemic control in T2D patients. However, whether herbal tea can integrate the advantages of herbal medicine and tea and play an ideal role in the treatment of T2D remains elusive.

As far as we know, some herbals are customarily made into tea to treat and prevent diseases. For example, *Hibiscus sabdariffa* L. originating in India is widely used in folk medicine in Asia, and the beverage made from the calyx of this plant is called Roselle tea, which has the function of regulating glucose and lipid metabolism^[25]^; Mate tea made from the dried leaves of *Ilex paraguariensis* is also a classical herbal tea in South America, and researches have also confirmed its metabolic regulating role by plenty of experimental and clinical researches.^[26]^ However, for the herbal tea with potential therapeutic roles on T2DM, there are no systematic reviews and meta-analyses to evaluate their efficacy and security, so our study can fill the gap. As for the selection of indicators, FBS is regarded as the most classical diagnostic indicator; while nowadays, more and more guidelines regard HbA1C as the more effective evaluation indicators, which can reflect the mean blood glucose level within 3 months.^[27]^ In addition, 2 hours PBG, FINS, and HOMA-IR can more comprehensively assess the improvement of blood glucose.

There are some potential limitations in our study. First, we only search English and Chinese databases, which will inevitably omit some researches. Second, there may exist a significant difference in efficacy among different kinds of herbal teas, and we will conduct subgroup analyses to discuss respectively if original studies are enough.

At last, we will publish our research findings in a peer-reviewed academic journal.

## Author contributions

**Conceptualization:** Boxun Zhang, Rensong Yue.

**Data curation:** Boxun Zhang, Xiaoying Huang.

**Formal analysis:** Boxun Zhang, Xiaoying Huang.

**Investigation:** Ying Wang, Yayi Jiang, Jiawei Chin.

**Methodology:** Boxun Zhang.

**Project administration:** Rensong Yue.

**Software:** Ying Wang, Yayi Jiang.

**Visualization:** Jiawei Chin.

**Writing – original draft:** Boxun Zhang.

**Writing – review and editing:** Rensong Yue, Jiawei Chin.
